# The Beta-Amyloid Protein of Alzheimer's Disease: Communication Breakdown by Modifying the Neuronal Cytoskeleton

**DOI:** 10.1155/2013/910502

**Published:** 2013-12-12

**Authors:** Sara H. Mokhtar, Maha M. Bakhuraysah, David S. Cram, Steven Petratos

**Affiliations:** ^1^Department of Anatomy and Developmental Biology, Monash University, Clayton, VIC 3800, Australia; ^2^Monash University, Clayton, VIC 3800, Australia; ^3^Department of Medicine, Central Clinical School, Monash University, Prahran, Clayton, VIC 3004, Australia

## Abstract

Alzheimer's disease (AD) is one of the most prevalent severe neurological disorders afflicting our aged population. Cognitive decline, a major symptom exhibited by AD patients, is associated with neuritic dystrophy, a degenerative growth state of neurites. The molecular mechanisms governing neuritic dystrophy remain unclear. Mounting evidence indicates that the AD-causative agent, **β**-amyloid protein (A**β**), induces neuritic dystrophy. Indeed, neuritic dystrophy is commonly found decorating A**β**-rich amyloid plaques (APs) in the AD brain. Furthermore, disruption and degeneration of the neuronal microtubule system in neurons forming dystrophic neurites may occur as a consequence of A**β**-mediated downstream signaling. This review defines potential molecular pathways, which may be modulated subsequent to A**β**-dependent interactions with the neuronal membrane as a consequence of increasing amyloid burden in the brain.

## 1. Introduction

Several neurodegenerative disorders share common characteristics including aggregation of misfolded mutant proteins in neurons leading to their deafferentation or loss with resultant structural or functional deficits in specific regions of the central nervous system (CNS) [1]. The most prevalent symptoms of age-related neurodegenerative disease are cognitive decline and movement disorders, along with brainstem and cerebellar signs. Such age-dependent disorders include Alzheimer's disease (AD), Huntington's disease (HD), Parkinson's disease (PD), and Spinal Cerebellar Ataxias (SCAs) [1]. There exists complexity in identifying fundamental molecular mechanisms precipitating neurodegeneration in these age-related brain diseases. However, common molecular signalling pathways have been defined in the specific neuronal populations associated with pathology [2]. Although the initiators of neuronal dysfunction may differ for each neurodegenerative disorder, there may be common molecular pathways which when being dysregulated, drive and exacerbate neurodegeneration. For example, the degeneration seen in AD is a result of amyloid plaques and phosphorylated tau deposition in the cerebral cortex and specific subcortical regions, leading to degeneration in the temporal lobe and parietal lobe, along with parts of the frontal cortex and cingulate gyrus [2]. AD also displays dysregulation in kinase and phosphatase mechanisms along with microtubule motor proteins during the degeneration phase [3, 4]. Therefore, a major question that remains unresolved is whether the dysregulation in specific kinases/phosphatases and vesicular transport mechanisms are aetiological contributors to AD pathology.

## 2. Neurodegeneration and Alzheimer's Disease (AD)

Over the past century, the ageing of our population (≥65 years) in industrialised countries has exceeded that of the population as a whole. It is predicted that in subsequent generations, the proportion of the elderly population will double and so will the proportion of persons suffering from neurodegenerative disorders [5]. Diagnosis of neurodegenerative disease is usually based on clinical symptoms as there are no suitable noninvasive tests that can specifically predict onset of these conditions. However, with the advent of specialised magnetic resonance imaging (MRI) techniques, it is now possible to detect early pathological changes in the brain [6], providing clinicians with a unique window for early therapeutic intervention. Nevertheless, it is imperative that biomarker(s) of neurodegeneration are identified to assist in the early detection of these idiopathic cognitive disorders. Such biomarkers may take the form of modified proteins or peptides that are released into the circulation or alternatively sequestered intrathecally [1, 2].

Biomarkers of neurodegeneration may well be derived from dysfunctional/modified proteins that form the basis of pathological signal transduction cascades [1]. The dysregulation of signalling molecules central for maintaining neuronal function may stimulate the onset of neurodegeneration. For example, while Rho kinase (mainly ROCKII), glycogen synthase kinase-3*β* (GSK-3*β*), cyclin-dependent kinase-5 (Cdk5), and phosphatases are all essential for normal neuronal development [7], they may all be involved in a plethora of neurodegenerative disorders through a central pathogenic mechanism.

## 3. Amyloid Beta (A***β***) and Amyloid Plaque Pathology

**Figure 1:**
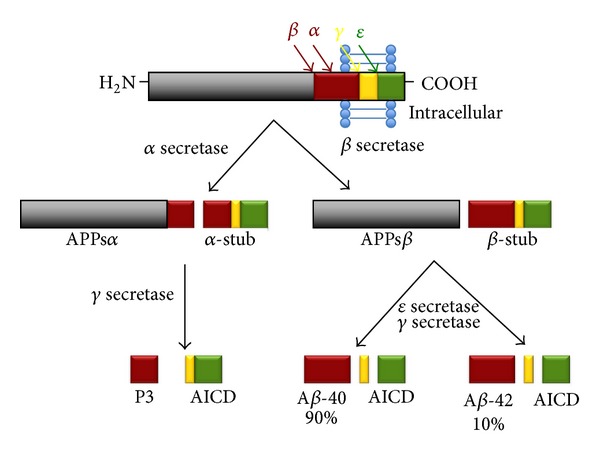
The processing of APP through the beta-site A*β*PP-cleaving enzyme BACE1, followed by presenilin-1 (PS1). Sequential beta and gamma-secretase cleavage of APP generates the synaptotoxic amyloid-*β* (A*β*) peptide species, A*β*1-40 and A*β*1-42.

It is well documented that the aging process is the major determinant of developing amyloid plaques with or without disease [8]. These extracellular senile plaques are composed of accumulated A*β* protein aggregating as *β*-pleated sheets and are derived from the aberrant cleavage of the transmembrane protein, APP [9–11]. Under normal physiological conditions, APP is a cell surface protein that is thought to be involved in signal transduction, axonal elongation, and cell migration [12–20]. It was also demonstrated that the C-terminus of APP plays a central role in gene expression and neuronal cell survival [21]. Such physiological mechanisms are only effective when APP is cleaved by various enzymes which can include intramembranous degradation by beta-site A*β*PP-cleaving enzyme (BACE1) to form the *β* C-terminal fragment (*β*CTF) [22, 23], subsequently followed by gamma-secretase which forms the small 4 kilodalton (kDa) amyloid-*β* (A*β*) peptides A*β*1-40 and A*β*1-42, which are released at the synapse (Figure 1) [22, 24, 25]. It has been demonstrated that the extent of APP cleavage is amplified in AD brains and that A*β* treatment further enhances this cleavage [21]. It has also been established that APP and its degradation products localise to neuritic vesicles [26] in the axons of AD brains, along with other neurodegenerative diseases, suggesting that APP accumulation may represent a hallmark of axonal injury [27, 28]. For instance, in APP transgenic mice, it has been demonstrated that elevated A*β* levels result in the loss of synapses and neuronal transmission along with behavioural abnormalities, before the formation of amyloid plaques [21]. Accumulation of A*β*, mainly A*β*1-42, results in the rare early-onset familial AD (EOFAD) which is caused by mutations in the enzymes that cleave APP, leading to rapid and aberrant cleavage with resultant overproduction of A*β* [29]. On the other hand, the common late-onset AD (LOAD) is thought to result from either the failure of A*β* to be cleared from the brain [30, 31] by microglial cells, lower expression of A*β* degrading proteases such as insulysin (insulin degrading enzyme IDE), a decline in the availability of A*β* chaperone low density lipoprotein receptor-related protein (LRP1) to transport A*β* out of the brain, reduced vascular and perivascular drainage, or a combination of the above [32]. Although A*β* monomers are relatively nonpathogenic, accumulating soluble A*β* oligomeric forms have been shown to be synaptotoxic and can prune dendritic spines, disconnecting the memory-encoding neuronal network in the entorhinal cortex, the parahippocampal gyrus, and the hippocampus [33]. These oligomers eventually form large insoluble fibrillar aggregates or plaques that by themselves do not directly induce neuronal death but rather attract microglia and astrocytes that produce cytotoxic proinflammatory cytokines and reactive oxygen species that may indirectly cause neuronal death [34].

Additionally, other proposed mechanisms that contribute to neuronal damage include the vulnerability of cells to secondary insults, tau hyperphosphorylation, induction of the apoptosome and lysosomal protease activity, changes in calcium influx, and direct damage (peroxidation) of membranes [35].

Although the plaques are found extracellularly, it is thought that production, oligomerisation, and accumulation of A*β* occur within neuronal processes with the possibility that the incorporation of aggregates into plaques occurs after the neurites are dissolved [36]. Certainly, studies performed in the well-established mouse models of AD have identified A*β* in several neuronal compartments such as the Golgi apparatus, the endoplasmic reticulum, the secretory vesicles, endosomes, and autophagic vacuoles, suggesting intraneuronal aggregation and pathology [36]. However, recent evidence supports the extracellular deposition of A*β* as the initiating pathogenic mechanism in the AD brain [37], with a direct correlation with the inhibition of anterograde axonal transport [38]. Despite direct evidence of A*β*-dependent neurodegeneration, A*β* pathology occurs prior to the appearance of clinical symptoms [37]. Accordingly, determining the level of amyloid deposition in an AD patient's brain (A*β* load) in a time-dependent manner would be informative in evaluating the progression of the disease and monitoring patient's response to antiamyloid therapies. Interestingly, through amyloid imaging, recent studies have demonstrated binding of the PET Pittsburgh compound B (PiB-PET) to A*β* peptides [39]. In this study, PET amyloid imaging with Pittsburgh compound B (PiB) showed increased cortical PiB binding in AD patients when compared to control subjects and intermediate binding levels in patients with mild cognitive impairment (MCI) [39]. This compound could be beneficial in the early detection of AD and evaluation of disease progression.

Recently, it was demonstrated by a combination of *in vivo* and *in vitro* studies that A*β* binding to the cellular prion protein (PrPc), an oligomer-specific high-affinity binding site for A*β*, can play a central role in A*β*-induced memory deficits, axon degeneration, synapse loss, and neuronal death in the AD brain through Fyn kinase activation [40]. The activation of this kinase results in alterations in N-methyl-D-aspartate receptor (NMDAR) function by increasing surface NMDAR expression along with its phosphorylation, and eventually leading to dendritic spine, in association with surface receptor loss [40]. The data suggests that by inhibiting PrPc in the APPswe/PSEN1-M146L double transgenic mouse, reversal of memory deficits and restoration of synaptic density could be achieved [40]. It has been demonstrated that Fyn kinase associates with the tau protein and that abnormal Fyn-tau interactions sensitise synapses to glutamate excitoxicity [40]. Together, these data suggest that PrPc-Fyn signalling may contribute to A*β* and tau pathologies and thus its downregulation may be a potential therapeutic approach.

## 4. Tau Protein Pathology

**Figure 2:**
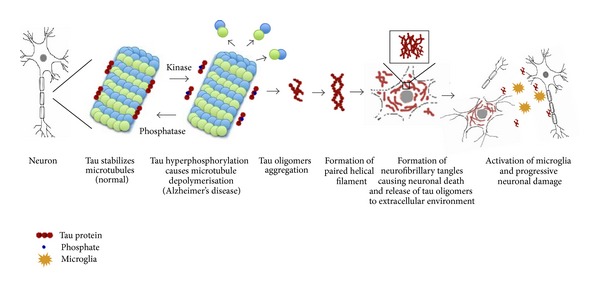
Stabilisation of microtubules by the tau protein is regulated by kinases and phosphatases. Abnormal hyperphosphorylation of tau proteins causes catastrophic microtubule depolymerisation and the formation of insoluble cytoplasmic tau oligomers, which aggregate to form protomers. Two protomers twisted around each other to form paired helical filaments (PHFs), which assemble to produce neurofibrillary tangles (NFTs).

The tau protein is an integral component of the neuronal cytoskeleton [41] with a molecular weight ranging from 45 kDa to 65 kDa [41] and is responsible for the promotion of microtubule assembly in the normal brain [42]. Microtubule assembly is tightly regulated by a combination of protein kinases and phosphatases that balance the amount of tau phosphorylation [43, 44]. The most common tau pathology is seen in AD, but it is manifest in other diseases such as frontotemporal dementias and Parkinson's disease [45, 46]. In the AD brain, tau exists in a hyperphosphorylated state, which leads to aberrant secondary structures and loss of function, resulting in a reduced ability to bind to microtubules and to promote their assembly [47]. The abnormal translocation of tau from axonal microtubules to neuropil thread inclusions, cell bodies and dendritic processes, where tau aggregates and accumulates, are pther prominent cytopathological hallmarks observed within AD brain sections [48]. The tau protein is initially synthesised as a single chain polypeptide and then targeted by posttranslational modifications that alter its conformation, promoting tau dimerisation in an antiparallel manner [49]. Stable tau dimers subsequently form tau oligomers, which aggregate at an increasing rate to form subunits of filaments, called protomers. Two protomers twisted around each other with a crossover repeat of 80 nm, constitute the width varying between w10 and w22 nm to form paired helical filaments (PHFs), a characteristic of AD neuronal pathology [49, 50]. Assembly of PHFs finally establishes the neurofibrillary tangles (NFTs), which can be observed microscopically (Figure 2) [51]. Hyperphosphorylated tau sequesters normal tau and other neuronal microtubule associated proteins (MAPs), such as MAP1A, MAP1B, and MAP2, contributing further to disassembled microtubules, disruption of the axonal cytoskeleton, and transport, culminating as damaged neurons [52]. After neuronal death, tau oligomers are released into the extracellular environment which leads to microglial cell activation and, as a consequence, further progressive bystander neuronal degeneration [53]. It has been suggested that tau pathology results from elevated protein kinase activity, a reduction in the activity of protein phosphatase, or both [45]. Analysis of phosphorylated tau isolated from AD brains has identified numerous target serine or threonine residues [45]. It has been demonstrated that MAP-kinase, GSK-3*β*, and/or Cdk5 are the main kinases involved in tau phosphorylation. However, in AD not all tau phosphorylation events can be attributed to these kinases [45].

The mechanism by which tau exerts its neuronal toxicity is still controversial [54]. It has been suggested that a series of degenerative signals such as A*β* aggregation, iron overload [55], oxygen free radicals [56], cholesterol levels in neuronal rafts, LDL species [57], and homocysteine can activate the innate immune response [53]. The activation of microglial cells, for instance, results in the subsequent release of pro-inflammatory cytokines that modify neuronal behaviour through anomalous signalling cascades, with the end result being the promotion of tau hyperphosphorylation [53]. However, numerous cellular and transgenic animal models indicate that tau is crucial for A*β*-induced neurotoxicity [54]. For instance, cultured hippocampal neurons from tau deficient mice are protected against A*β* pathology [54]. Furthermore, in cultured hippocampal neurons from wild-type mice, the silencing of tau by siRNA has demonstrated that tau is required for prefibrillar A*β*-induced microtubule disassembly. Furthermore, it was demonstrated that a reduction in soluble A*β* and tau but not A*β* alone causes cognitive decline in the triple transgenic AD mouse model with plaques and tangles [58]. These data suggest that although A*β* is the initial trigger, tau accumulation plays a central role in neurodegeneration. Finally, in the AD-like transgenic model that expresses human APP with familial mutations, suppression of endogenous tau prevented A*β*-dependent water maze learning and memory deficits without reversing the amyloid pathology [58]. Collectively, these data suggest a link between A*β* and tau that drive the neural pathologies and the manifestations of clinical symptoms. Preliminary data on the inhibition of tau aggregation by methylene blue chloride (MTC) has indicated a lower rate of cognitive decline in treated patients compared with those sporadic AD patients on alternate therapies, implicating tau as the key initiator of cognitive deficits [50]. However, the exact role of A*β* dependent in signal transduction cascades that are associated with pathogenic tau modifications and the contribution to the progression of neuronal death require further investigation [54].

## 5. Signalling Molecules Linked with Neuronal Cytoskeleton Disassembly

### 5.1. Rho Kinase (ROCK)

The Rho-associated coiled-coil forming protein kinases (ROCKs) include the ROCK-1 and ROCK-2 isoforms. These two kinases contain highly conserved aminoterminal but different carboxy-terminal domains [59]. Both ROCK-1 and ROCK-2 were originally shown to be involved in cell differentiation, essential for the regulation of myogenesis from embryonic fibroblasts along with skeletal muscle maturation and differentiation [60]. Both Rho kinase (ROCKs) and p21-activated kinase (PAKs) are members of the serine/threonine class of protein kinases. However, they are known to have antagonistic effects on the actin cytoskeleton and therefore on the plasticity of synapses. PAK also has two major isoforms, PAK1 and PAK3, and they have downstream signalling effects on Rho/Rac (for review see [61]). PAK can stimulate actin polymerisation [62], axon outgrowth, and the formation of dendritic spines [63] through LIM kinase stimulation [62]. PAK can also inhibit the myosin light chain kinase (MLCK) which diminishes actomyosin contractility [64, 65].

It has been reported that 13-month-old AD-like mice (PDAPP) displayed a substantial decrease in PAK 1-3 activity compared to normal controls [21]. Furthermore, the hippocampi of patients exhibiting the early clinical signs of AD have displayed high PAK 1-3 activity which was then shown to decline in the late stages of AD pathology [21]. It was further suggested that C-terminal cleavage of APP at the Asp664 site mediates PAK abnormalities and that an Asp664 mutation may potentially prevent these abnormalities [21]. On the other hand, ROCKs stimulate the retraction of axonal and dendritic growth cones by activating MLCK through the phosphorylation of myosin light chain proteins to promote an interaction with actin [66]. In addition, ROCK2 can phosphorylate collapsin response mediator protein 2 (CRMP2), another microtubule associated protein, to induce growth cone collapse [67].

Moreover, many developmentally or pathologically regulated molecules can also activate the RhoA/ROCK pathway to inhibit axonal growth including semaphorins, ephrins, and myelin inhibitory factors, such as Nogo and myelin-associated glycoprotein (MAG). On the other hand, there are some signalling molecules such as Sema4D/plexin-B1 that activate the RhoA/ROCK pathway especially in hippocampal neurons that may induce dendritic spine formation. It has been speculated that this may be due to the activation of LIM kinase and the PAK-type response via actin-depolymerising factor ADF/cofilin [68, 69].

In AD, dendritic spine defects play a major role in cognitive impairments [61]. It has been reported that dendritic postsynaptic proteins are excessively distorted with disease progression [70]. For instance, neuronal loss in the hippocampi of AD patients is approximately 5–40% while the loss of postsynaptic proteins such as the developmentally regulated actin-regulating brain protein (drebrin), which is targeted by A*β* oligomers, reaches 70–95% [71]. This study in particular suggested that A*β*-induced alteration in postsynaptic PAK may have a central role in the massive drebrin loss and cognitive deficits found in AD, which could be prevented by an antibody to A*β* and/or by *in vivo* or *in vitro* overexpression of wild-type PAK [71].

**Figure 3:**
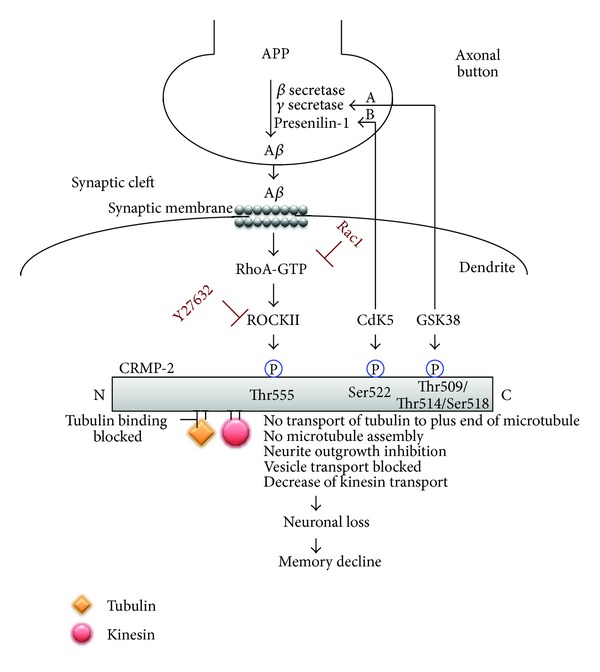
Model of A*β*-mediated neurite outgrowth inhibition. A*β* (oligomeric) activates the small GTPase, RhoA, which inhibits the proneurite outgrowth GTPase Rac1. RhoA-GTP activates Rho Kinase (ROCK II) to effect microfilament rearrangement and also potentiate microtubule disassembly. Microtubule disassembly occurs when ROCK II directly phosphorylates CRMP-2 at the Thr555 position preventing the association of CRMP-2 with tubulin heterodimers, thereby affecting neurite outgrowth inhibition. Neurite outgrowth is further impeded by CRMP-2 phosphorylation since this prevents the microtubule motor protein, kinesin, to associate with CRMP-2 and transport growth-related vesicular cargo, such as BDNF, antergradely to the distal end of the neurite. It is demonstrated that CRMP-2 is also phosphorylated by GSK-3*β* and Cdk-5. (A) Studies have demonstrated that GSK-3*β* activity can also regulate the processing of APP resulting in the production of A*β*, which in turn can further increase GSK-3*β* activity through PI3K inhibition, illustrating as a potential feedback loop. (B) Additionally, it has been suggested that Cdk5 may phosphorylate presenilin-1 at Thr354 destabilising its carboxy-terminal fragment, leading to increased APP processing.

Cognitive defects and eventually dementia are important clinical features of AD (for review see [2]). It has been reported that there exists a relationship between the cognitive-decline occurring in AD along with genetic mental retardation syndromes and synaptic dysfunction, primarily since the postsynaptic maintenance of dendritic spines is lost. To maintain synaptic balance, both ROCK1 and 2 transduce signals to retract the growth cones and dendritic spines (for review see [72]). It has been shown that ROCK may provoke APP breakdown to the toxic *β*-amyloid 1-42 peptide. For example, ROCK inhibitors, such as Y27632, inhibit the toxic processing of APP [73]. An intriguing conundrum is that the binding of A*β* on neurons may activate RhoA and ROCK2 to potentiate the phosphorylation of its substrates [74, 75]. One of the specific substrates that our group has recently defined is CRMP-2 (Figure 3). It has been shown that CRMP-2 exhibits hyperphosphorylation in the cortex of AD postmortem brains [76]. Experimentally, it has been illustrated that other kinases such as GSK-3 and Cdk5 can also phosphorylate CRMP-2 and produce growth cone collapse in neurons [77] (Figure 3). Our data suggest that *β*-amyloid can increase the RhoA-GTP level in differentiated SH-SY5Y cells increasing CRMP-2 phosphorylation and reducing the neurite lengths in cultured neuroblastoma cells. Additionally, RhoA and CRMP-2 levels are elevated in neurons surrounding amyloid plaques in the cerebral cortex of the APP (Swe) Tg2576 AD mouse model. Our work indicates that A*β* induces Rho GTPase activity and ROCK2 to promote CRMP-2 phosphorylation which can lead to the inhibition of neurite outgrowth [78] (Figure 3). However, a direct link with the reduction of ROCK2-dependent CRMP-2 phosphorylation and the limitation of cognitive decline is yet to be established in the context of A*β*-dependent neurodegeneration.

### 5.2. Glycogen Synthase Kinase-3*β* (GSK-3*β*)

The proline-directed serine/threonine kinase, glycogen synthase kinase-3 (GSK-3), is important for several cellular processes such as metabolism, cell structure, and apoptosis and in the regulation of gene expression (for review see [79]). The GSK-3 family contains two members, GSK-3*α* and GSK-3*β*, that are highly expressed in the brain and spinal cord with GSK-3*β* playing a central role in neuronal differentiation and the maintenance of neurons (for review see [80]). Activation of GSK-3 requires prephosphorylation by other priming kinases such as Cdk5 at serine or threonine sites located 4 residues, C-terminal to the site phosphorylated by GSK-3 (for review see [81]) (Figure 3). Abnormal GSK-3 function has been implicated in different brain pathologies indicating its fundamental role in controlling basic mechanisms of neuronal function, modulation of neuronal polarity, migration, proliferation, and survival, not to mention the establishment of neuronal circuits (for review see [82]). It has been demonstrated that phosphorylation of GSK-3 may influence cytoskeletal proteins altering neuronal plasticity (for review see [83]). Neuronal cytoskeletal changes occur due to an altered rate in the stabilisation/destabilisation of microtubules (MT), thereby altering the dynamics of dendrites, spines, axons, and synapses. Intensified efforts in the identification of enzymes involved in regulating tau phosphorylation *in vivo* have revealed GSK-3*β* as a candidate kinase for therapeutic targeting [79] during AD pathology.

It has been hypothesised that GSK-3 overactivity may potentiate sporadic and familial forms of AD by enhancing tau hyperphosphorylation [84] and APP processing and possibly through the phosphorylation of CRMP-2 leading to profound memory impairment [81] (Figures 2 and 3). It has been established that the expression of full-length unmodified or unphosphorylated CRMP-2, in primary hippocampal neurons or SH-SY5Y neuroblastoma cells, promotes axon elongation. Moreover, cultured neurons expressing CRMP-2 with mutant GSK-3 phosphorylation sites (T509A, S518A) display significantly reduced axon elongation [81]. On the other hand, studies have demonstrated that GSK-3*β* phosphorylation of the CRMP-2 T509 site can play a crucial role in mediating the repulsive action of Sema3A [85] and promoting growth cone collapse [77]. Recently, Cole et al. have demonstrated that dephosphorylation of CRMP-2 at the GSK-3*β*-dependent sites (Ser-518/Thr-514/Thr-509) can be carried out by a protein phosphatase 1 (PP1) *in vitro*, observed in neuroblastoma cells and primary cortical neurons, and that the inhibition of GSK-3*β* by insulin-like growth factor-1 or the highly selective inhibitor CT99021 results in dephosphorylation of CRMP-2 at these sites [86]. How this may be translated to real therapeutic outcomes during AD pathology is yet to be demonstrated, even within animal models of disease.

### 5.3. Cyclin-Dependent Kinase-5 (Cdk5)

The other proline-directed serine/threonine kinase, identified as a major priming enzyme for tau phosphorylation, is cyclin-dependent kinase-5 (Cdk5) [87]. Although Cdk5 is ubiquitously expressed in most tissues, it is not directly involved in mediating progression through the cell cycle as it requires prior activation by p35 and p39, which are expressed almost exclusively in the CNS [88]. Cdk5 plays an important role in CNS development possibly by mediating interactions between neurons and glia during radial migration, which is essential for developing appropriate cortical laminar architecture [89, 90]. Furthermore, Cdk5 has been reported to also play a role in neuronal differentiation, axonal guidance, synaptic plasticity, cellular motility, cellular adhesion, and neurodegeneration (for review see [91]).

Studies have shown that inhibition of Cdk5 reduces A*β*-induced neurodegeneration in cortical neurons [92] which highlights that targeting Cdk5 could be a future therapeutic strategy for neurodegenerative disorders. The critical microtubule associated protein, CRMP-2, has been also demonstrated to be a substrate for Cdk5 [77]. This study showed an orderly phosphorylation process of CRMP-2 by Cdk5 (defining it as the priming kinase) followed by GSK-3*β* as a consequence of Sema3A stimulation that inhibits axonal growth [77]. Alternatively, a non-phosphorylated form of CRMP-2 cannot respond to Sema3A signalling. This study also demonstrated that Sema3A promotes phosphorylation of CRMP-2 at Ser522, which is the established Cdk5 phosphorylation site [77]. Thus, targeted kinase inhibitors may possibly be therapeutically beneficial in AD to limit both tau and CRMP-2 phosphorylation. Deciphering which of the kinases precipitate neurodegeneration is still under investigation but when elucidated, the possibility exists that formulation of specific inhibitors to prevent cognitive decline associated with AD is achievable.

### 5.4. Phosphatases

Protein phosphatases provide unique endogenous signalling mechanisms for the dephosphorylation of proteins, reversing such posttranslational modifications, which may limit protein dysfunction. Protein phosphatase 2A (PP2A) is one of the most important serine/threonine phosphatases in the mammalian brain. It also exists in most tissues comprising up to 1% of total cellular protein. It has major roles in development, cell growth, transformation (for review see [3]), regulation of protein phosphorylation, and cell signalling pathways [93]. PP2A is composed of 3 subunits: subunit A (scaffolding/structural), subunit B (regulatory/targeting), and subunit C (catalytic) [94]. PP2A with PP1 collectively account for more than 80% of the total serine/threonine phosphatase activity in all mammalian cells [3, 95] making these enzymes integral to cellular physiology.

*In situ*, PP2A, PP1, PP5, and PP2B account for 71%, 11%, 10%, and 7%, respectively, of the total tau phosphatase activity in the human brain [96]. PP2A is the most prevalent phosphatase involved in tau dephosphorylation [97]. Knockdown of PP2A phosphatase activity was shown to lead to tau hyperphosphorylation [98]. Furthermore, when PP2A was inhibited in cultured cells and in transgenic mice with mutant PP2A, hyperphosphorylation of tau was observed [98]. Moreover, the naturally abundant SET protein, a potent PP2A inhibitor, is found to be elevated in AD brains [99], possibly illustrating reduced PP2A activity allowing for the hyperphosphorylation of cellular substrates to occur unabated and the potentiation of neurodegeneration. Interestingly, autopsy studies of brains from AD patients, non-AD dementia, and normal human brains demonstrate that there is loss in PP2A protein, mRNA, and enzymatic activity in areas of the brain affected by AD, the hippocampus and cortex, but not in the cerebellum [100]. In addition, the inhibition of PP2A activity mimics most of the phosphorylation events seen in AD, such as tau hyperphosphorylation [101].

Phosphorylation of APP by an array of kinases has been shown to influence its cleavage by *β*-secretase resulting in A*β* production [102]. It was demonstrated that PP2A has the ability to dephosphorylate APP at the Thr668 site and thus inhibit A*β* generation [103]. Studies of cells expressing the (APPswe) mutation, transgenic mice expressing both APPswe and presenilin mutations, and sections of hippocampus and entorhinal cortex from human AD patients, show that PP2A levels are decreased and Y307 levels (an inhibitor of PP2A) were increased [104] implying that the phosphatase affects the processing of APP and highlighting its importance in limiting AD pathology. In N2a cells, where PP2A was inhibited with okadaic acid (OA), the phosphorylation of APP and the secretion of both sAPP*α* and sAPP*β* were all elevated [105]. In addition, inhibition of the protein phosphatases PP1 and PP2A in rat brain by OA results in the accumulation of hyperphosphorylated tau and A*β* species [45, 94]. Even though incubation of different types of cells with OA resulted in the stimulation of APP secretion, it was not proven that the effect was mediated by PP1 [106] and/or PP2A [107]. Moreover, it was demonstrated that demethylation of PP2A by nuclear phosphatase methylesterase-1 (PME-1) reduces its activity and thus leads to tau hyperphosphorylation along with APP phosphorylation, promoting APP cleavage and A*β* production [108–110]. Collectively, these results suggest that downregulation of PP2A may induce A*β* production and tau phosphorylation, precipitating AD pathology.

A direct link of PP2A activity with the progression of AD pathology has been affiliated to the fact that CRMP-2 phosphorylation may actually be a result of lowered PP2A activity [93]. Since CRMP-2 hyperphosphorylation was commonly observed to correspond with progressive neurodegeneration, decreased PP2A may well regulate such a disease-specific event. However, such a hypothesis would need to be substantiated beyond a causal link.

### 5.5. Collapsin Response Mediator Protein (CRMP)

The collapsin response mediator proteins (CRMPs) are members of the dihydropyrimidinase-related neuronal phosphoprotein family [111]. The CRMP family has five isoforms, CRMP1-5 [112]. The most well characterised of these, CRMP-2, is highly expressed in the adult mammalian CNS localising in the cytoplasm and neurites of postmitotic neurons [111]. CRMP-2 is also highly expressed in the areas of the adult brain of greatest plasticity such as the hippocampus, olfactory bulb, and cerebellum [113]. In neurons, CRMP-2 is concentrated within the distal portions of neurites, in synapses and in growth cones [114]. It regulates the polarity and differentiation of neurons through the assembly and trafficking of microtubules [115]. CRMP-2 has no known enzymatic activity by itself but through an interaction with other binding partners it can regulate neural differentiation, dendrite/axon fate specification, Ca^2+^ homeostasis, neurotransmitter release, regulation of cell surface receptor endocytosis, kinesin-dependent axonal transport, growth cone collapse, neurite outgrowth, and microtubule dynamics (for review see [78, 116]). The last three functions have been demonstrated to be regulated by phosphorylation near the C-terminus of CRMP-2 by kinases [117, 118] including cyclin-dependent kinase 5 (Cdk5), glycogen synthase kinase-3*β* (GSK-3*β*) [31, 76, 86, 119], Tau-tubulin kinase-1 (TTBK1) [120], and Rho kinase II (ROCKII) [78, 117, 121], all of which culminate in neurite retraction (for review see [78]). CRMP-2 hyperphosphorylation in AD was suggested to be a result of increased kinase activity, decreased phosphatase activity, or both [86]. All phosphorylation events can disrupt the association of mature full-length CRMP-2 with tubulin heterodimers possibly resulting in the destabilisation of the neuronal microtubule system rendering axonal retraction [67]. Moreover, disruption of the binding between CRMP-2 and tubulin due to the phosphorylation of CRMP-2 can block tubulin transport to the plus ends of microtubules for assembly (Figure 3) [78], blocking neurite outgrowth/elongation. In primary neurons and neuroblastoma cells, it has been demonstrated that overexpression of CRMP-2 results in axon elongation [114] while overexpression of truncated CRMP-2, lacking the C-terminus tubulin binding domain, inhibits axon growth. These data implicate this region of CRMP-2 to play a central role in axonal growth [114]. Both the Cdk5 and GSK-3*β* phosphorylation of CRMP-2 have been shown to be increased in the cortex and hippocampus of the triple transgenic mouse (PS1/APP/Tau mutant), along with the double transgenic mouse (PS1/APP mutant), that develop AD-like plaques along with NFTs. However, in transgenic mice, which display only mutant tau (P301L) that develop tangles but do not develop amyloid plaques, Cdk5 phosphorylation of CRMP-2 does not occur. These results indicate that hyperphosphorylation of CRMP-2 might be induced by APP overexpression and/or its enhanced processing, thereby generating a high amyloid load within the brain of these transgenic mice [76].

Our laboratory has recently demonstrated that, in human neuroblastoma SH-SY5Y cells and in the Tg2576 mouse model of AD, A*β* can reduce the length of neurites by inactivating the neurite outgrowth-signalling molecule Rac1 [78]. Furthermore, the data suggested that A*β*-mediated reduction in neurite length could be reversed by the Rho Kinase inhibitor (Y27632). Additionally, the A*β*-mediated decrease in neurite length was linked to the promotion of a threonine phosphorylation of CRMP-2 (unrelated to GSK-3*β*-dependant phosphorylation), conferring a reduced binding capacity to tubulin, both of which can be reversed by inhibiting RhoA activity [78]. These data suggested that A*β*-mediated neurite outgrowth inhibition results from the activity of RhoA-GTP and the dysregulation of CRMP-2 to bind tubulin for neurite outgrowth [78] (Figure 3).

Studies using transgenic mouse models expressing the Swedish familial AD mutant (APP/TTBK1) demonstrated that the induced upregulation of tau tubulin kinase-1 (TTBK1) can promote axonal degeneration via phosphorylation of CRMP-2 and tau within the entorhinal cortex and hippocampus, implicating TTBK1 as a potential therapeutic target for AD [120].

Despite the profound link to CRMP-2-dependent degeneration through kinase-mediated phosphorylation, another function of CRMP2 is mediated through its known association with kinesin, facilitating the anterograde molecular transport of growth promoting vesicles along axonal microtubules [122]. The exact mechanism of binding and transport and its contribution to AD will be discussed in detail below.

## 6. CRMP2-Tubulin Binding

The microtubule and actin cytoskeleton orchestrates axonal growth cone dynamics by a process of signal transduction leading to either depolymerisation or polymerisation events, for directional growth [119]. As already discussed above, the binding of CRMP2 to tubulin heterodimers can enhance microtubule assembly leading to axon outgrowth [123, 124]. Semaphorin-3A (Sema3A) is an extracellular protein that can block axonal outgrowth [77] through the activation of Cdk5, with downstream phosphorylation of both tau and CRMP-2 [31, 77]. Such phosphorylation can disrupt their tubulin association limiting axonal growth. Following the Cdk5 phosphorylation of CRMP-2, the latter may potentiate a conformational change leading to subsequent phosphorylation by GSK-3*β* [31, 77]. However, it has been demonstrated that in GSK-3*β* overexpressing mice, no hyperphosphorylation of CRMP-2 can be identified at the GSK-3*β* phosphorylation sites and furthermore phosphorylation of tau does not increase [125]. This may explain the finding that activation of GSK-3*β* alone can not induce growth cone collapse (for review see [119]). Interestingly, protein lysates from human AD cortex and animal models of AD show hyperphosphorylation of CRMP-2 at residues Thr509, Thr514, and Ser518 which are known to be the GSK-3*β* phosphorylation sites as well as Ser522, the well-known Cdk5 phosphorylation site (for review see [78]). These findings indicate that Sema3A signalling may regulate microtubule polymerisation through the physiological actions of tau and CRMP-2, which regulate the dynamics of microtubules and tubulin dimers, respectively [126]. Phosphorylation of CRMP-2 by Rho kinase at the Thr555 site, however, can also reduce the CRMP-2 association with tubulin heterodimers and induce growth cone collapse unrelated to Sema3A signalling and quite possibly be the result of A*β*-dependent signalling [31, 77]. The phosphorylation of CRMP-2 by Cdk5, GSK-3*β*, and Rho kinase may therefore play a central role in coordinating cytoskeletal activities in response to multiple axon guidance cues [31, 77].

The plausible hypothesis exists that activation of all thre kinases Cdk5/GSK-3b/ROCK2, contribute to the destabilisation of the neuronal microtubule system in AD. Consequently, tau and CRMP-2 have some similarities in that both control microtubule polymerisation and stability and they both respond to the growth cone guidance molecule Sema3A [77]. Therefore, it can be theorised that a balanced treatment which may successfully decrease CRMP-2 phosphorylation could also be effective in regard to tau aggregation and vice versa in AD (for review see [31]).

## 7. Microtubules (MT)

One of the most important physiological features of the multipolar neuron is to have a polarised axon, that can extend to more than 1 meter in the human CNS [127]. For the neuron to function normally, it should be able to transport vital molecular cargo from its body to synaptic terminals and vice versa in a timely manner through the axon via anterograde and retrograde transport mechanisms, respectively [127, 128]. Therefore, it stands to reason that the integrity of the microtubule transport system is crucial for axonal transport [129]. The microtubule system facilitates ATP driven transport through molecular motors of the cell's vital components which include vesicles, proteins, mitochondria, chromosomes, and large macromolecules such as microtubule heterodimers themselves [128, 130]. The transport machinery directly interacts with microtubules and includes two families of proteins categorised according to their directional movement. These proteins include either microtubule plus end-directed kinesins or the microtubule minus end-directed cytoplasmic dynein [127].

Many neurodegenerative diseases, such as AD, display a blockade in microtubule transport, emphasising its significance in normal physiology and highlighting abnormal neuronal vesicle trafficking as a potential pathogenic mechanism [130–132]. It is believed that A*β* may cause mitochondrial dysfunction and, therefore, axonal transport defects [132]. It has been demonstrated that APP processing and A*β* overproduction in the mitochondria lead to mitochondrial dysfunction and therefore reduction of mitochondrial energy supply and inhibition of axonal transport [133]. Enhancing energy supply of neurons could be critical to compensate for the A*β*-dependent loss of energy and thus facilitate axonal transport.

Microtubule depolymerisation has been touted as a contributing factor in the gross loss of memory, as it is necessary to stabilise newly formed microtubules in spines for long-lasting memory [134, 135]. There exists evidence implicating tubulin sequestration [136] and blockade in microtubule assembly as a pathogenic mechanism of AD [129]. It has been recently demonstrated that *in vitro*, microtubules can be assembled from the cytosol of normal autopsy brain obtained within five hours postmortem, while this is not possible from identically treated AD postmortem brain tissue [129]. Furthermore, it has been documented that axonal transport is defective in neurons from AD postmortem brains indicating the destruction of the microtubule cytoskeleton in axons of diseased neurons [134]. There also exist data suggesting that the abnormality in axonal transport might stimulate the formation of, or enhance the accumulation of, A*β* [134, 137], through autophagocytosis of mitochondria without normal lysosomal degradation [137].

One of the main physiological functions of tau is to stimulate microtubule assembly by polymerising with tubulin, maintaining the microtubule structure and stability through its capacity to anchor polymerised microtubules to the internal axolemma [129]. Evidence for the role of tau and microtubule destabilisation arises from tau transgenic mice which show spinal cord tau inclusions [131]. In this animal model, an inability of tau to stabilise microtubules can be compensated with the MT-stabilising agent paclitaxel resulting in increased MT density and marked improvement in motor function [131]. However, paclitaxel is thought to have poor blood-brain barrier permeability and thus is an unlikely candidate for human therapy during neurodegeneration [131].

In the early stages of AD pathogenesis, observations within the neuropil demonstrate that there exists an abnormal aggregation of the activated actin-associated protein cofilin, a protein that modulates actin-rich dendritic spine architecture, which is important for learning and memory [43]. Those neuropil threads can disrupt the cytoskeletal network by blocking cargo trafficking to synapses, resulting in memory and cognition impairment [43]. It is also suggested that abnormal activation of cofilin may trigger the accumulation of phosphorylated tau in neuropil threads [43]. The activities of cofilin and the protein actin-depolymerising factor (ADF) are regulated by phosphorylation and dephosphorylation through LIM and other kinases, along with chronophin phosphatases, respectively [43]. Heredia et al. found that *β*-amyloid may activate LIMK1 and thus stimulate ADF/cofilin phosphorylation in cultured neurons [69]. Moreover, they demonstrated, in the AD brain, that the number of P-LIMK1-positive neurons was extensively increased in the affected regions [69]. A recent study of AD transgenic mice demonstrated that neuronal cell bodies are viable although the neurites are damaged [138]. Taken together, these studies highlighted that the development of *in vivo* methods to disrupt LIMK1 activation, the formation of the cofilin-actin rods, and/or the interaction between cofilin and pMAP, may be a plausible way to stop the disease early in its presentation.

## 8. Kinesin

The microtubule motor protein complex, kinesin-1, has a fundamental role in the vesicular transport from the neuronal cell body, along the axon and anterograde, to the synapse (for review see [139]). The motor protein complex consists of two kinesin heavy chains (KHC) that have both an ATP and the microtubule binding motif which are essential for vesicle transport [140]. Two kinesin light chains (KLC) that associate with the heavy chain and vesicular cargo membranes [140] complete the structure of the transport protein. APP is one of the molecular candidates for receptors that attach kinesin-1 to vesicular cargo [139]. The carboxy terminus of APP binds directly to the light-chain subunits of kinesin-1 [140] and thus plays a major role in the recruitment of kinesin-1 to axonal vesicles [141]. Moreover, the level of axonal APP is suggested to play a central role in determining expression levels of kinesin-1 decorating vesicles, providing the ability to determine the anterograde movement behaviour of APP-containing vesicles [141]. It has been reported that kinesin blockade and axonal swellings are involved in the pathogenesis of the early stages of AD even before the formation of amyloid plaques and neurofibrillary tangles, although the initiating events are not clear [142]. Moreover, in animal models, *β*-amyloid formation and its subsequent transport are enhanced when kinesin transport is abrogated or impaired [38, 141]. Axonal transport damage results in the development of axonal swellings where APP is processed into smaller A*β* species. APP axonal transport is mediated by direct binding to KLC1 [143]. Genetic manipulation designed to damage APP axonal transport in AD mouse models, such as Tg-swAPP^Prp^, demonstrated the enhancement in the incidence of axonal swellings, elevated A*β* levels, and potentiated the production of amyloid deposition [142]. In particular, APP directly interacts with KLC1 (the microtubule transport machinery) through its carboxy terminus, suggesting that impaired interaction of APP and KLC1 might play a central role in the AD pathogenesis [144]. Decreased KLC1 transport may also stimulate tau hyperphosphorylation and formation of NFTs as well as axonal swellings producing catastrophic damage to axons. Such damage may arise from increased A*β* levels and tau hyperphosphorylation, further disrupting axonal transport [145].

It is now well established that CRMP-2 plays a central role in negotiating fast axonal transport by acting as an adaptor protein to the microtubule motor kinesin-1, for propagation of anterograde vesicle transport of key traffic molecules such as the high affinity neurotrophin receptor, tyrosine kinase (TrkB). Following distal localisation of this receptor, TrkB is inserted into the cell membrane and activated by its cognate ligand brain-derived neurotrophic factor (BDNF), resulting in axonal growth through signalling within the growth cone, thereby establishing the accumulation and polymerisation of F-actin and tubulin. In AD, phosphorylated CRMP-2 releases kinesin-1, inhibiting TrkB function and limiting the structural integrity of the actin-based cytoskeleton in distal axons, growth cones, and synapses [146]. Inhibiting CRMP-2 phosphorylation could be beneficial to restore tubulin and kinesin-1 binding to CRMP-2 and thus promoting axonal outgrowth and transport of important molecular cargo.

## 9. Conclusion

Alzheimer's disease (AD) is an age-related progressive neurodegenerative disorder and is the most common form of dementia in the elderly. The hallmarks of AD pathology are the extracellular deposition of a 4 kDa amyloid beta (A*β*) polypeptide and the formation of intracellular neurofibrillary tangles (NFTs) along with dystrophic neurites, degenerating neurons, and activated astrocytes and microglia, a part of the reactive pathology observed around senile plaques. Neuritic plaques result from the aggregation of the amyloid *β* protein (A*β*) which is a consequence of amyloid precursor protein (APP) aberrant processing. The corresponding accumulation of filamentous inclusions within the CNS as neurofibrillary tangles (NFTs), resulting from the hyperphosphorylation of the microtubule-associated protein, tau and amyloid deposition, are both pathognomonic to sporadic AD. There is an impressive list of genes and proteins involved in AD pathologies including APP, presenilins, secretases, kinases, and phosphatases all touted as being responsible for either increasing the production of the neurotoxic A*β* protein or promoting the hyperphosphorylation of CRMP-2 or tau, leading to the devastating neurodegenerative sequelae. The understanding of the major gene players cooperating with key environmental factors that contribute to the manifestation of AD pathology is fundamental in the derivation of a more comprehensive understanding of AD pathogenesis and for the development of specific and more effective treatments of this devastating age-dependent disease.
